# Pediatric respiratory syncytial virus rehospitalization rate – a retrospective observational study from Switzerland

**DOI:** 10.1186/s12887-025-05887-z

**Published:** 2025-07-12

**Authors:** Naomi Rupp, Nina Schöbi, Andrea Duppenthaler, Carmen Casaulta, Matthias V Kopp, Philipp KA Agyeman, Christoph Aebi

**Affiliations:** 1 Division of Pediatric Infectious Disease, Department of Pediatrics, Bern University Hospital, Inselspital, University of Bern, Bern, CH-3010 Switzerland; 2 Division of Pediatric Respiratory Medicine, Department of Pediatrics, Bern University Hospital, Inselspital, University of Bern, Bern, Switzerland; 3 Airway Research Center North (ARCN), University of Lübeck, Lübeck, D-23562 Germany

**Keywords:** Respiratory syncytial virus, RSV, Rehospitalization, Readmission, Nirsevimab, Vaccine, Prophylaxis

## Abstract

**Background:**

Long-acting monoclonal antibodies against Respiratory Syncytial Virus (RSV) have recently become available for prevention of severe disease including RSV hospitalization in children below two years of age. Data on the risk of rehospitalization among children, who had suffered from severe first RSV episode, remain important to inform the need for secondary prevention using a (additonal) dose of such an antibody. We studied the risk of RSV rehospitalization in a large cohort of patients with a particular focus on same-season rehospitalizations.

**Methods:**

Retrospective single-center study of all RSV rehospitalizations occurring in 13 RSV seasons between 2009 and 2023 based on an ongoing RSV surveillance program. We calculated the overall and same-season rates of rehospitalizations for patients of any age and for the first 5 years of life, respectively, and provide a clinical description of of rehospitalization cases.

**Results:**

In a cohort of 3’143 patients having had a primary RSV hospitalization, the overall risk of rehospitalization (69 cases) and same-season risk of rehospitalization (2 cases) for a second RSV infection were 2.2% (95% confidence interval (CI), 1.73–2.79) and 0.06% (95% CI 0.02–0.23), respectively. The figures for the RSV rehospitalization rates from birth until age 5 years of age were 2.3% (95% CI 1.76–3.07) for all rehospitalizations and 0.04% (95% CI 0.01–0.25) for same-season rehospitalizations. The median length of stay (LoS) of rehospitalizations (4.0 days, interquartile range (IQR) 3.0–6.0) was significantly shorter than the LoS of first hospitalizations (6.0 days, IQR 4.0–9.0, *p* < 0.0001). Children with a pre-existing condition (68%) and those born prematurely (40%) predominated among rehospitalized patients.

**Conclusion:**

Same-season RSV rehospitalizations were exquisitely rare. Routine administration of a dose of a monoclonal antibody for protection against a same-season rehospitalization does not appear to be generally warranted. The majority of patients with subsequent season readmission would be covered by the current recommendations in Switzerland as they had pre-existing conditions making them eligible for second-season RSV prophylaxis.

**Supplementary Information:**

The online version contains supplementary material available at 10.1186/s12887-025-05887-z.

## Background

Respiratory Syncytial Virus (RSV) is the leading cause of acute lower respiratory tract infection necessitating hospital admission in children below five years of age worldwide [1]. Approximately 70% of the annual birth cohort in temperate climates experience their primary infection with RSV in the first year of life [2–5], and 1–3% are hospitalized for acute RSV bronchiolitis or other lower respiratory tract disease [6–8]. Several host factors increase the risk of severe disease. These include premature birth, male sex, chronic lung disease of various etiologies, hemodynamically significant heart disease, several other comorbidities, crowded living conditions, and parental smoking [8–10]. The most important risk factor in absolute numbers, however, is young chronological age in otherwise healthy infants [8, 9, 11].

In 2023, the first extended-half-life monoclonal antibody for passive immunization of children below two years of age [12] and the first active vaccine for maternal immunization in late pregnancy [13] became available for prevention. Both provide protection against clinically relevant disease including RSV hospitalization in the range of 70–90% for at least 5 months [13–18]. These agents have the potential to dramatically reduce the overall disease burden, but RSV hospitalizations in infants and toddlers will continue to occur. Their frequency will depend on vaccination rates, their real-world effectiveness, the epidemiology of a given RSV season [8], and the interval between vaccination and exposure to the virus.

Therefore, a clinical question arising is whether children who have suffered a virologically documented RSV infection – either because they or their mother had previously been unimmunized or as a breakthrough disease despite immunization – should be offered a (additional) dose of a monoclonal antibody for protection during the ongoing RSV season. To inform how such a recommendation should be formulated, it is necessary to quantitate the risk of severe reinfection, best assessed by calculating the risk of RSV rehospitalization. Only a few studies have addressed this question to date [19–21] Studies from Europe [20, 21] are several years old and covered short observation periods only. The purpose of the present study was thus to generate new data on the rate of RSV rehospitalizations in a European country covering an extended period of time and to describe their clinical characteristics.

## Methods

### Settings and case catchment

This is a single-center, retrospective study of all patients admitted to the in-patient-service of the Department of Pediatrics, Bern University Hospital, between 1 July 2009 and 30 June 2023, focusing on those with two or more hospitalizations associated with a virologically confirmed RSV infection. Our institution is the only provider of pediatric in-patient-services for a general population of 1.1 million and an average annual birth cohort from 2009 to 2023 of 9’722 [22]. General hospitals without pediatric departments in Switzerland do not accept patients below 16 years age. The annual RSV season begins in November and ends in April. There is a biannual periodicity with major and minor seasons alternating [8, 23]. In was interrupted by the COVID-19 pandemic, which resulted in a near complete suppression of the 2020–2021 season [8, 23]. RSV surveillance based on a screening program for RSV from a nasopharyngeal sample obtained in the emergency room of all patients admitted with an acute respiratory disease was in place during the entire study period [8, 23].

### Identification of rehospitalization cases

Cases of RSV rehospitalization were identified by searching the RSV hospitalization database for identical unique personal identification (PID) numbers that each patient receives at first contact in this institution and keeps throughout all subsequent encounters. Cases were considered as matching if the following three criteria were identical: PID, date of birth, first and/or last name.

### Case definition

RSV hospitalization was defined as a hospital stay on an in-patient ward extending over at least one date change. RSV rehospitalization was defined as a given patient’s second or subsequent hospitalization for a RSV positive acute lower respiratory tract infection (LRTI), if it occurred at least 30 days after discharge from the previous RSV hospitalization [19]. RSV associated LRTI was defined in accordance with the WHO case definition for severe or very severe RSV LRTI [24]. Virologic confirmation of RSV infection consisted of the detection of RSV in a nasopharyngeal sample obtained on the day of admission using a direct immunofluorescence assay [25], quantitative RNA Polymerase Chain Reaction (PCR) or rapid antigen detection assay [26]. Study inclusion required parental written informed consent for the scientific use of routine chart data (general consent).

### Variables and analysis

We extracted the following variables from each case record: date of birth, sex, admission and discharge dates, RSV-related main diagnosis as listed in the discharge summary, gestational age, single or multiple pregnancy, pre-existing conditions including chronic respiratory conditions and their specific type, hemodynamically significant heart disease, neurologic and neuromuscular disorders, Down syndrome, renal diseases, immunodeficiency, other pre-existing conditions, and intensive care unit (ICU) admission. These variables were analyzed for all cases combined as well as for both primary and secondary RSV hospitalizations separately. The rehospitalization rate was calculated in two ways. (1) The crude rate was established by dividing the number of rehospitalizations by the number of all RSV hospitalizations occurring during the entire study period. (2) The rehospitalization rate for the first 5 years of life was established by calculation rehospitalization rates in under-5-year-old patients born between 1 July 2009 and 30 June 2018. This allowed to ascertain that all patients included were followed up to the age of 5 years.

### Statistical analyses

Data are presented as frequencies and proportions for categorical variables and as medians and interquartile ranges (IQR) for continuous variables. Categorical variables were compared with the χ2 test or Fisher Exact test. Continuous variables were compared with the Mann–Whitney U-test or the Kruskal-Wallis test. Demographic data were obtained from the Federal Office of Statistics [22]. VassarStats (http://vassarstats.net) and GraphPad Prism version 10.0.0 for Windows (GraphPad Software, Boston, Massachusetts USA) were used for analyses and figure drawing.

## Results

**Fig. 1:**
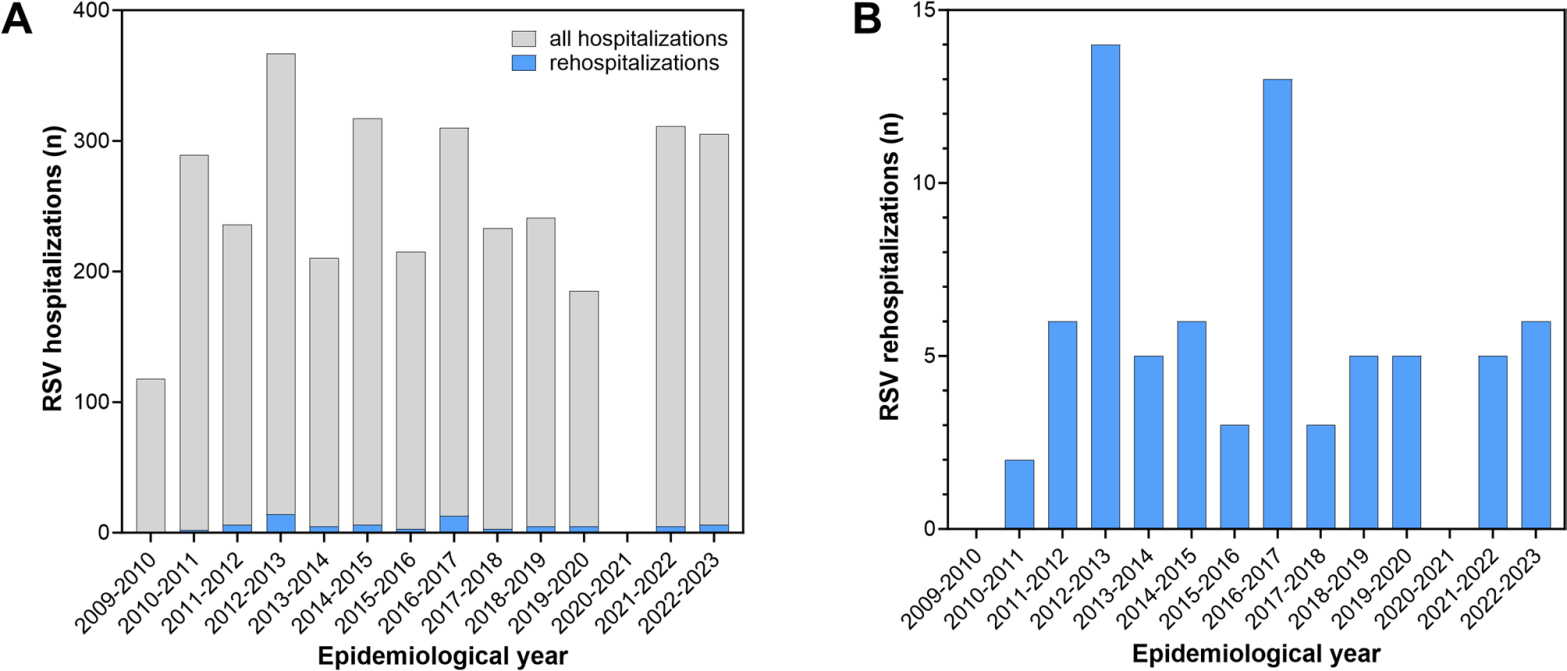
Time sequence of annual RSV hospitalization frequencies in each epidemiological year from 2009 to 2023. In panel **A**, the gray bars indicate the total number of RSV hospitalizations per year, the blue bars denote the number of rehospitalizations in each year. Panel **B** shows rehospitalizations only

**Fig. 2:**
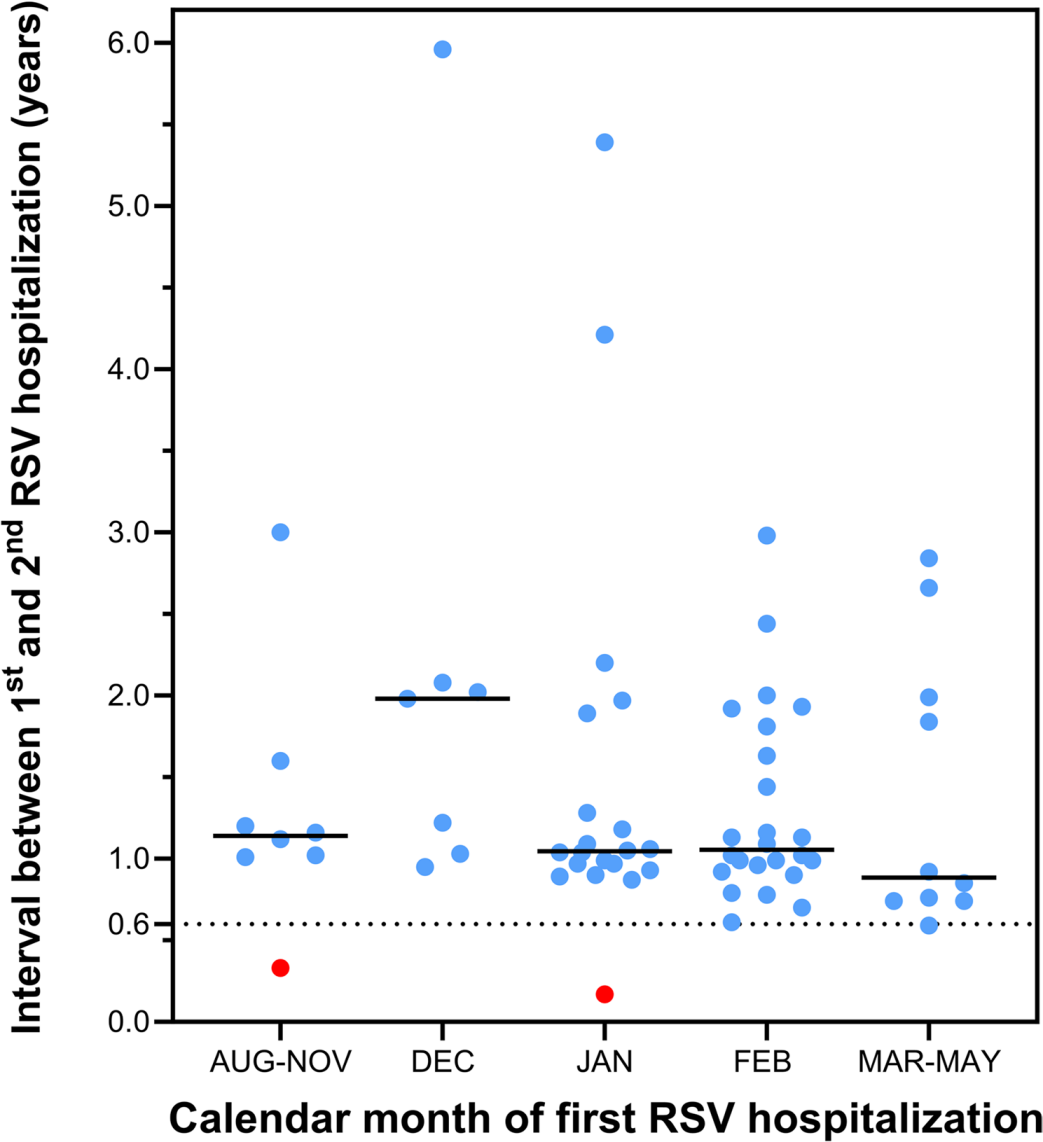
Intervals in years between the first and the second RSV hospitalization in relation to the calendar month, in which the first hospitalization occurred. Each dot identifies one patient. The red dots identify the two cases with same-season rehospitalization. The dotted lane marks the minimum interval found among the remainder of patients

In-house RSV surveillance recorded a total of 3’358 RSV hospitalizations occurring in 3’282 patients during the 14-year observation period. The latter encompassed 13 RSV seasons as winter 2020–2021 saw no cases. The flow chart in Figure S1 (supplementary data file) illustrates how the study cases were identified and how the study cohort was recruited. Following exclusion of 142 cases occurring in 139 patients, whose parents declined consent for the use of routine clinical data, we identified 3’216 hospitalizations in 3’143 patients as the denominator cohort. Of these, 69 patients were rehospitalized with a RSV infection at least once, yielding a crude rehospitalization rate of 2.2% (95% confidence interval (CI), 1.73–2.79). Figure 1 depicts the annual case frequencies of all RSV hospitalizations and of RSV rehospitalizations during the observation period from 2009 to 2023. For calculation of the under-5-year rehospitalization rate we identified a denominator cohort of 2’231 patients born between 1 July 2009 and 30 June 2018, among which 52 rehospitalizations occurred (rehospitalization rate 2.3%, 95% CI, 1.76–3.07). The median interval between the first and second RSV hospitalization was 1.1 years (interquartile range (IQR), 0.9–1.9, Fig. 2).

Two of 69 patients (3.0%) were rehospitalized during the same RSV season as the first hospitalization. Clinical details of these cases are given in Table S1. Thus, two of the total of 3’143 patients with a first RSV hospitalization were rehospitalized during the same season (0.06%, 95% CI 0.02–0.23). In the under-5-year cohort, one of a total of 2’231 patients was rehospitalized during the same season (0.04%, 95% CI 0.01–0.25). Sixty-seven second hospitalizations occurred later (44 (60%) in the subsequent season, 23 (32%) in a later season). A third RSV hospitalization was recorded in 4 patients (6%). The length of hospital stay (LoS) of first hospitalizations was significantly longer than the LoS of second hospitalizations (Table 1). ICU admissions were somewhat more frequent during the first hospitalization (10 cases (14.5%) vs. 6 cases (8.7%); *p* value, 0.246). Two patients (3.0%) required ICU care in both their first and second hospitalization.

**Table 1:** Characteristics of patients with RSV rehospitalizations admitted between 1 July 2009 and 30 June 2023 to the department of pediatrics, Bern university hospital, bern, Switzerland

Characteristic	Denominator	*n* (%)/median [range]
Patients		69
Male sex	69	39 (57)
Rehospitalizations		
2nd hospitalizations	69	69 (100)
3rd hospitalizations	69	4 (6)
Age, years		
1st hospitalization		0.6 [0.3–1.1]
2nd hospitalization		1.8 [1.4–3.1]
3rd hospitalization		4.4 [4.2–7.1]
Gestational age, weeks		
≥37	60	36 (60)
32–36	60	15 (25)
<32	60	9 (15)
Timing of rehospitalization		
same RSV season	69	2 (3)
subsequent RSV season	69	45 (65)
later RSV season	69	22 (32)
Length of hospital stay, days		
1st hospitalization		6.0 [4.0–9.0]^1^
2nd hospitalization		4.0 [3.0–6.0]^1^
3rd hospitalization		5.0 [3.8-7.0]
Intensive Care Unit admissions		
1st hospitalization	69	10 (14)^2^
2nd hospitalization	69	6 (8.7)^2^
3rd hospitalization	4	0
Pre-existing condition		
None	69	22 (32)
Chronic respiratory tract condition	69	42 (61)
Congenital heart disease, hemodynamically significant	69	5 (7.2)
Down syndrome	69	5 (7.2)
Neuromuscular disease	69	4 (5.8)
Immunodeficiency	69	3 (4.3)
Renal failure	69	2 (2.9)
Seizure disorder	69	2 (2.9)
Received monthly palivizumab	69	0

^1^*p* = 0.0001 (Mann-Whitney U-test)

^2^*p* = 0.426 (Fisher Exact test)

Twenty-four of 60 patients (40%), whose gestational age (GA) at birth was known, were former premature infants (Table 1). The risk of rehospitalization in the pediatric population served by our institution was inversely related to GA. Children born at < 32 weeks and 32–36 weeks had 23-fold and 7-fold greater rehospitalization risk, respectively, than children born at term (Table S2). Analysis of pre-existing conditions revealed that a respiratory tract disease was noted in the majority of rehospitalized patients (61%). The list of respiratory comorbidities encountered is shown Table 2. Episodic viral wheeze or multiple trigger wheeze were the most common conditions (31 patients). In 22 patients, one of these two diagnoses had already been established by the time of rehospitalization. In 9 patients, it was first noted in the patients’ medical record at a later time only.

**Table 2:** Chronic respiratory tract conditions among 69 patients with RSV rehospitalizations

Patients and conditions	*n* (%)
All patients with RSV rehospitalizations	69 (100)
Chronic respiratory tract condition	42 (61)
Episodic viral or multiple trigger wheeze^1^	31 (45)
Bronchopulmonary dysplasia	4 (6)
Anatomic anomaly^2^	4 (6)
Cystic fibrosis	1 (1)
Multifactorial	2 (3)

^1^known at the time of RSV rehospitalization in 22 patients

^2^Lung hypoplasia (2 cases), bronchial malformations (2 cases)

We next examined factors potentially associated with the length of the interval between RSV hospitalizations. Table 3 compares clinical characteristics of subsequent-season vs. later-season rehospitalizations. No major differences were detected. Figure 2 shows the intervals between each hospitalization for all 69 patients, sorted according to the age at first RSV admission. Younger chronological age at the first RSV admission was not associated with a shorter time to rehospitalization. For instance, the median time [IQR] to rehospitalization for patients < 6 months vs. 6–11 months vs. ≥12 months, respectively, did not significantly differ (1.1 [1.0–2.0] vs. 0.9 [0.8–1.4] vs. 1.2 [1.0-1.9] years; p value 0.173, Kruskal-Wallis-test). Further, patients with severe pulmonary, cardiac or neuromuscular comorbidities (Fig. 2, blue dots) tended to be older at the time of their first RSV admission, but the median time [IQR] to rehospitalization did not differ significantly from those without such comorbidity (1.1 [1.0–2.0] vs. 1.0 years [0.9–1.2]; *p* value 0.139). This was also the case for the presence vs. absence of any respiratory tract condition (1.1 [1.0–2.0] vs. 1.0 years [0.9–1.2]; *p* value 0.234).

**Table 3:** Comparison of subsequent-season vs. later-season RSV rehospitalizations

Characteristic	subsequent-season		later-season		Odds ratio (95% confidence interval) or *p* value	
No. of patients (n)	45		26^1^			
Male sex, n (%)	28 (62)		13 (50)		1.65 (0.62–4.37)	
Median age at first hospitalization, years [IQR]	0.5 [0.3–0.9]		0.9 [0.3–1.3]		0.529	
Median age at rehospitalization, years [IQR]	1.5 [1.3–1.9]		3.6 [2.8–4.9]		< 0.0001	
Gestational age < 37 weeks, n (%)^2^	16 (39)		10 (39)		0.88 (0.33-2-40)	
Pre-existing condition, n (%)	28 (62)		22 (85)		0.30 (0.09–1.02)	
Median length of hospital stay, days [IQR]	4.0 [3.0–5.0]		4.5 [3.0–7.0]		0.263	
Intensive Care Unit admissions, n (%)	3 (6.6)		3 (12)		0.55 (0.10–2.93)	

^1^includes the 4 cases of 3rd hospitalizations

^2^gestational age was available in 40 cases (subsequent season) and 20 cases (later season), respectively

**Fig. 3:**
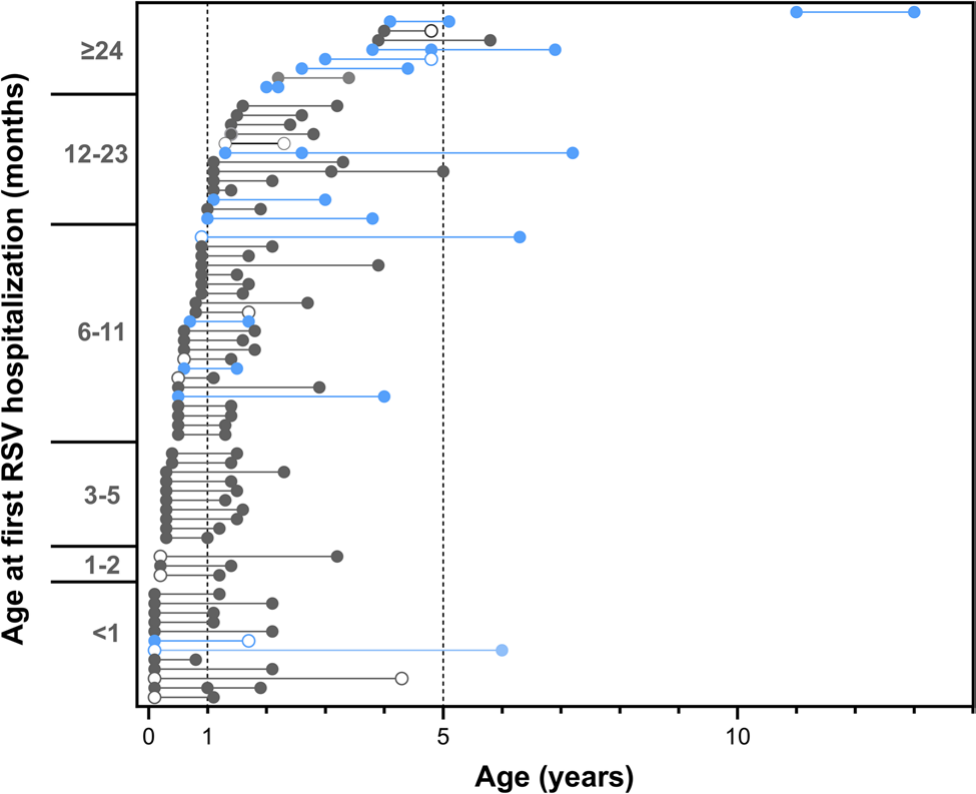
Intervals (in years on the x-axis) between each RSV hospitalization are shown for each of the 69 patients with RSV rehospitalization(s) included in the study. Each dot denotes the patient age at an RSV hospitalization. Open circles identify ICU admissions. On the y-axis, patients are sorted from bottom to top according to their chronological age in months at the time of the first admission. Blue dots and lines identify patients with a severe pre-existing condition (pulmonary, cardiovascular, neuromuscular)

Finally, we examined the relationship between the seasonal timing of the first RSV hospitalization and the interval to rehospitalization. Figure 3 demonstrates that the later in the season the first RSV hospitalization occurred, the shorter the minimal interval to readmission. Except for the two same-season readmission cases (red dots), the minimal interval recorded was 0.6 years (7.2 months) and it occurred in patients hospitalized for the first time in February or later.

## Discussion

The data of this long-term retrospective study identify an overall RSV rehospitalization rate of 2.2%, and a very low same-season rehospitalization rate of 0.06%. The vast majority of rehospitalizations occurred in patients between one and 5 years of age, were generally milder and of shorter duration than the primary RSV hospitalization, and occurred predominantly in children with respiratory tract comorbidities and/or with former premature birth.

Several previous reports examining the RSV rehospitalization rate are available for comparison. In Austria, Resch and co-workers found a rehospitalization rate of 1.9% among 745 children admitted for a RSV infection over a 6-year period [20]. Ten of 14 affected patients had a risk factor for severe RSV disease, which included former prematurity in 6 cases. In a study from Italy, Barbati et al. found a RSV rehospitalization rate of 0.8% among 624 patients hospitalized over a 5-year period [21]. In a large Canadian study comprising 10’212 cases of RSV hospitalizations in children below 5 years of age over a 13-year period, Wang et al. reported a rehospitalization rate of 2.3% [19]. Former prematurity below 37 weeks and congenital heart disease were identified as risk factors independently associated with an elevated rehospitalization risk.

Thus, the rehospitalization rate found in our study is somewhat greater than in the two neighboring countries [20, 21], but the difference is of minor clinical relevance. Possible causes include varying case identification and RSV hospitalization criteria, respectively, and different case definitions for RSV rehospitalization, which were not stated in these studies. Our findings, however, were nearly identical to those reported in the Canadian study [27], which covered a similarly long observation period. We used the same definition for readmission as specified in this report, i.e. an interval > 30 days between first admission discharge and rehospitalization.

Same-season rehospitalizations are a subgroup of particular importance in the new era of immunoprophylaxis with extended half-life monoclonal antibodies [12, 28]. For an infant diagnosed with an RSV infection, who is either unimmunized or infected despite immunoprophylaxis, the question arises whether an (additional) dose of a monoclonal antibody for protection during the ongoing season is to be recommended or not. We found an extremely low same-season rehospitalization rate of 0.06%, reflecting two out of 3’143 patients. Both had atypical case histories as their first hospitalization occurred after infancy. Wang et al. identified 36 cases resulting in a same-season rehospitalization rate of 0.35% [27], roughly 6 times greater than our figure, but still very low. While we observed no cases of former prematurity among same-season readmissions, Wang et al. found an increased risk for premature children, which was, however, non-significant compared with term-born children [27]. Based on our finding, the current Swiss recommendation for the use of nirsevimab [28], which stipulates that nirsevimab is generally not indicated for children, who already had a documented RSV infection in the same season, appears justified. This is in line, e.g., with the recommendations in France [29] or Germany [30], but not the USA, where nirsevimab is recommended irrespective of a previous RSV infection [31].

We found that 40% of all RSV rehospitalizations occurred in children born at < 37 weeks of GA. Based on the annual birth cohorts of the hospital’s catchment area and population, we calculated a 9.9-fold increased risk for RSV rehospitalization associated with former prematurity (table S2). Similarly, in the Canadian study, prematurity was identified as an independent risk factor for RSV rehospitalization [27]. With respect to this finding, the Swiss guideline recommends second-season RSV immunoprophylaxis for children born < 32 weeks of gestation, but not for those born at 32–36 weeks [28]. Listing former late premature children with a previous-season RSV hospitalization among the indications for second-season prophylaxis is thus worth a consideration, but would only benefit a very small number of children. In Spain, e.g., second-season nirsevimab is recommended for former premature infants below 35 weeks who are less than 12 month of age at the beginning of the season [32]. In the USA, on the other hand, second-year prophylaxis is not recommended on the basis of prematurity alone [33].

Two-thirds of all cases in our series occurred in patients with a comorbidity. Except for the large number of those with recurrent wheezing, the pre-existing conditions encountered in our study are listed as indications for second-season immunoprophylaxis [28].

Precise data on the minimum duration of protection afforded by a primary RSV infection with need for hospitalization against a severe reinfection again requiring admission are difficult to generate. The interval between two hospitalizations is a composite of the duration of immune protection, the time to re-exposure and the host’s susceptibility for severe disease. RSV reinfection of any severity is common during the second RSV season of life. In a study from Finland [4], the rates for a first reinfection in the second or third year of life, respectively, among children first infected in infancy have been estimated at 30–40% in both years, while a third infection (i.e., second reinfection) until 3 years of life was rare (1%). In a study from the USA, the reinfection rate in the second year of life was 75% [5]. Our findings demonstrate that rehospitalizations may occur as soon as seven months (0.6 years) after the first admission, when the latter occurred late in the previous season. On the other hand, protection lasted for at least 5–6 months, because early-season first hospitalizations apparently protected for the remainder of the season. It remains open whether this period of protection reflects solely the patients’ immune protection or whether the immunity of close contacts co-infected around the time of the patients’ first admission substantially contributed to this effect. Importantly, there was no correlation between the chronological age at first hospitalization and the time to readmission.

Limitations of this study include (1) the single-site design, which may limit the validity of the findings for other locations although RSV tends to cause relatively uniform clinical manifestations and our findings corroborate those from another large study [27]. (2) Our findings underestimate the true figures, because some patients may have moved away from the hospital’s catchment area between two RSV hospitalizations. However, population mobility data from the Federal Office of Statistics [22] indicate that a stable 2% of the resident population of the catchment area moved away per year. Thus, the loss of children who had moved away between the first and a second RSV hospitalization is expected to be low. (3) Same-season rehospitalizations were so rare that the confidence interval of the rehospitalization rate remains wide. (4) We currently lack clinical evidence that RSV infection in patients previously immunized (monoclonal antibody or maternal vaccination) results in an immune response as robust as after infection without previous immunization. What we know is that infants immunized with nirsevimab do rise an immune response upon exposure to RSV [3] and that they do not appear to be more susceptible to RSV disease in their second RSV season than unimmunized infants [34].

In summary, this study confirms previous studies from Canada and two neighbouring countries of Switzerland that RSV rehospitalizations in children are infrequent, and that same-season rehospitalizations are exquisitely rare. The data support current recommendations to generally not administer a dose of a monoclonal antibody to children with a previous, virologically documented RSV infection during the same RSV season.

## Supplementary Information

Supplementary Material 1.

## Data Availability

The dataset generated in the process of project is available from the corresponding author on reasonable request.

## Abbreviations

CI: Confidence interval
GA: Gestational age
ICU: Intensive care unit
IQR: Interquartile range
LoS: Length of stay
LRTI: Lower respiratory tract infection
PCR: Polymerase chain reaction
PID: Patient identification number
RNA: Ribonucleic acid
RSV: Respiratory syncytial virus
