# Supplementary material for: Pediatric respiratory syncytial virus rehospitalization rate – a retrospective observational study from Switzerland
Source: BMC Pediatr. 2025 Jul 12;25:550. doi: 10.1186/s12887-025-05887-z (PMC12255118; doi:10.1186/s12887-025-05887-z)
Supplement: Supplementary file 1 — Supplementary Material 1. [file 12887_2025_5887_MOESM1_ESM.docx]

**Pediatric Respiratory Syncytial Virus rehospitalization rate – a retrospective observational study from Switzerland**

Naomi Rupp, Andrea Duppenthaler, Nina Schöbi, Carmen Casaulta, Matthias V Kopp, Philipp KA Agyeman, Christoph Aebi

**Supplementary data file**

**Figure S1**

Flow chart detailing case identification and selection of the study population of patients with RSV rehospitalizations admitted to the Department of Pediatrics, Bern University Hospital, Bern between 01 July 2009 and 30 June 2023.


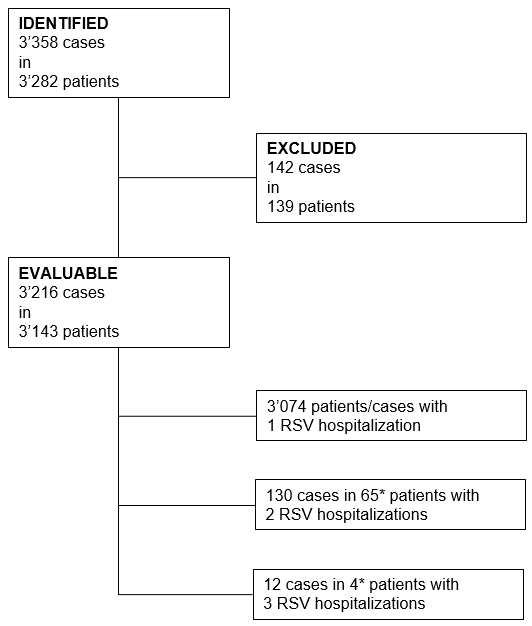


*the study group consists of these 69 patients with a total of 73 rehospitalizations.

**Table S1**. Clinical summary of the two cases of same-season rehospitalization

| Variable | Case 1 | Case 2 |
| --- | --- | --- |
| Sex | male | male |
| Pre-existing condition | Cystic fibrosis | no |
| Gestational age (weeks) | not known | 38 3/7 |
| Age at 1st hospitalization (years) | 2.0 | 1.1 |
| Interval between 1st and 2nd hospitalization (days) | 61 | 119 |
|  |  |  |
| RSV detection method 1st hospitalization | immunofluorescence | immunofluorescence |
| RSV detection method 2nd hospitalization | immunofluorescence | rapid antigen test |
|  |  |  |
| Viral co-infection 1st hospitalization^1^ | no | no |
| Viral co-infection 2nd hospitalization^1^ | no | rhino-enterovirus |
|  |  |  |
| Length of stay 1st hospitalization (days) | 5 | 2 |
| Length of stay 2nd hospitalization (days) | 17 | 7 |
|  |  |  |
| Main diagnosis 1st hospitalization | wheezy bronchitis | wheezy bronchitis |
| Main diagnosis 2nd hospitalization | wheezy bronchitis | wheezy bronchitis |
|  |  |  |
| Supplemental O_2_ administration 1st hospitalization | no | low-flow |
| Supplemental O_2_ administration 2nd hospitalization | low-flow | high-flow |
|  |  |  |
| Antibiotic therapy 1st hospitalization | yes | no |
| Antibiotic therapy 2nd hospitalization | yes | no |
|  |  |  |
| ICU admissions | no | no |
| ^1^nasopharyngeal specimens were tested for RSV, influenza A, influenza B, parainfluenza type 1-3, human metapneumovirus, rhino-enterovirus, adenovirus | | |

**Table S2. Population-based risk of RSV rehospitalization according to gestational age (GA) at birth**

|  | No. of patients with RSV rehospitalizations  2009-2023 (n) | |  |  |  |
| --- | --- | --- | --- | --- | --- |
| Gestational age (GA) | GA known | GA extrapolated* | Birth cohort^2^ 2009-2023 (n) | Risk of rehospitalization (%) | OR (95% CI) vs. GA ≥37 wks |
| <32 weeks | 9 | 10 | 1’359 | 0.762 | 23.0 (11.5-45.9) |
| 32-36 weeks | 15 | 17 | 7’474 | 0.231 | 7.1 (4.0-12.4) |
| <37 weeks | 24 | 28 | 8’833 | 0.312 | 9.9 (6.1-15.9) |
| ≥37 weeks | 36 | 41 | 127’052 | 0.033 | 1 |
| all | 60 | 69 | 135’885 | 0.051 | - |
| ^1^ the GA being known in 60 of 69 patients only, we extrapolated the case figures for each GA  accordingly. | | | | | |

^2^ obtained from the Federal Office of Statistics (www.bsf.admin.ch)
